# MICAL-L1 is required for cargo protein delivery to the cell surface

**DOI:** 10.1242/bio.058008

**Published:** 2021-06-08

**Authors:** R. Sikora, P. Bun, L. Danglot, M. Alqabandi, P. Bassereau, F. Niedergang, T. Galli, A. Zahraoui

**Affiliations:** 1 Université de Paris, Inserm U1016-CNRS UMR 8104, Institut Cochin, Paris, France; 2 Université de Paris, Institute of Psychiatry and Neuroscience of Paris (IPNP), INSERM U1266, Membrane Traffic in Healthy & Diseased Brain, Paris, France; 3 Université de Paris, Institute of Psychiatry and Neuroscience of Paris (IPNP), INSERM U1266, NeurImag Imaging facility, 75014 Paris, France; 4 Laboratoire Physico Chimie Curie, Institut Curie, PSL Research University, CNRS, UMR168, 75005, Paris, France; 5 GHU PARIS psychiatrie & neurosciences, F-75014 Paris, France

**Keywords:** Rab effectors, MICAL-L1, Membrane traffic, Membrane tubules

## Abstract

Secreted proteins are transported along intracellular route from the endoplasmic reticulum through the Golgi before reaching the plasma membrane. Small GTPase Rab and their effectors play a key role in membrane trafficking. Using confocal microscopy, we showed that MICAL-L1 was associated with tubulo-vesicular structures and exhibited a significant colocalization with markers of the Golgi apparatus and recycling endosomes. Super resolution STORM microscopy suggested at the molecular level, a very close association of MICAL-L1 and microdomains in the Golgi cisternae. Using a synchronized secretion assay, we report that the shRNA-mediated depletion of MICAL-L1 impaired the delivery of a subset of cargo proteins to the cell surface. The process of membrane tubulation was monitored *in vitro*, and we observe that recombinant MICAL-L1-RBD domain may contribute to promote PACSINs-mediated membrane tubulation. Interestingly, two hydrophobic residues at the C-terminus of MICAL-L1 appeared to be important for phosphatidic acid binding, and for association with membrane tubules. Our results reveal a new role for MICAL-L1 in cargo delivery to the plasma membrane.

## Abstract (precis)

**Summary:** MICAL-L1, an effector of Rab GTPases, exhibits a significant colocalization with markers of the Golgi apparatus and recycling endosomes. It is involved in cargo delivery to the plasma membrane.

## INTRODUCTION

Membrane trafficking is a tightly regulated process required for plasma membrane (PM) homeostasis, adhesion, and cell polarity. Secreted cargoes are transported from the endoplasmic reticulum to the Golgi apparatus (GA) and are then addressed to the plasma membrane. Small GTPase Rab proteins and their effectors regulate membrane trafficking between different intracellular compartments (Stenmark, 2009; Wandinger-Ness and Zerial, 2014). Rab8 plays a role in the exocytosis of AP-1B-dependent cargo (Ang et al., 2003). It is also required for the outgrowth of the primary cilium, and regulates apical protein localization in intestinal cells (Nachury et al., 2007; Sato et al., 2007; Yamamura et al., 2008). In addition, Rab8 and molecule interacting with CasL 3 (MICAL3) cooperate in controlling the docking and fusion of exocytotic carriers (Grigoriev et al., 2011). Rab13 regulates tight junction assembly by controlling the delivery of tight junction proteins and downregulation of PKA activity (Köhler et al., 2004; Marzesco and Zahraoui, 2005; Morimoto et al., 2005)*.* It also regulates membrane trafficking between GA and PM (Nokes et al., 2008).

Molecule interacting with CasL-like1 (MICAL-L1), was identified as an effector of several Rabs including Rab 8, 11, 13 and 35 (Abou-Zeid et al., 2011; Giridharan et al., 2012; Kobayashi et al., 2014; Sharma et al., 2009; Zahraoui, 2014). MICAL-L1 has a calponin homology (CH), a LIM (Lin-1l, Isl-1 and Mec-3), a proline rich (PRD) and a Rab binding domains (RBD) that binds GTP-bound Rab proteins. MICAL proteins contain an N-terminal flavoprotein monooxygenase domain (FAD) involved in F-actin oxidation and disassembly (Grigoriev et al., 2011; Hung et al., 2013). However, MICAL-L1 and 2 sequences do not contain the FAD. MICAL-L1 regulates endocytic recycling and plays a role in ciliogenesis (Sharma et al., 2010, 2009; Xie et al., 2019). In this study, we found that MICAL-L1 is partially associated with markers of the Golgi apparatus and recycling endosomes. We then investigated whether MICAL-L1 regulates the biosynthetic delivery of membrane proteins. To avoid non physiological temperature conditions and to monitor trafficking of different cargo proteins, the RUSH (retention using selective hooks) system was used (Boncompain et al., 2012). The RUSH assay allows the retention of a tagged cargo of interest in the endoplasmic reticulum (ER) and then the cargo is released following the addition of biotin in the cell media. This method proved to be very powerful to quantitatively assess the secretion of several cargoes (Boncompain et al., 2012; Boncompain and Weigel, 2018; Chen et al., 2017). We used the RUSH assay to characterize MICAL-L1 function in transport of membrane proteins to the PM. Depletion of MICAL-L1 experiments suggest that the protein is required for surface delivery of a subset of cargo proteins. Our data further indicate that two amino acids at MICAL-L1 C-terminus are important for phosphatidic acid (PA) binding and association with membrane tubules.

## RESULTS

### MICAL-L1 is associated with Golgi like structure and with cytoplasmic tubulo-vesicles

**Fig. 1.**
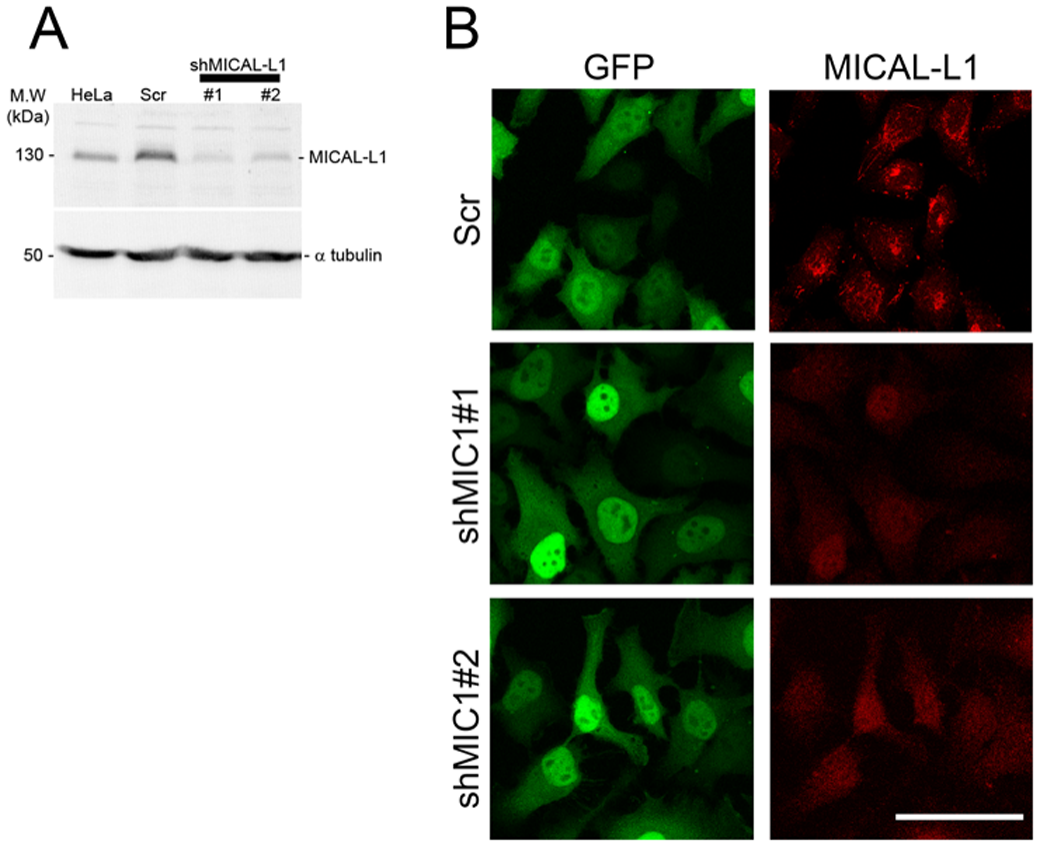
**Anti-MICAL-L1 antibodies specifically recognize the protein**. (A) Equal amounts of proteins (30 µg) prepared from HeLa cells and from HeLa expressing scramble (Scr) and two shRNAs targeting MICAL-L1 were analyzed by immunoblot using affinity purified anti-MICAL-L1 antibodies. Tubulin was used as loading controls. (B) HeLa cells on coverslips expressing Scr and shRNAs targeting MICAL-L1 were fixed and incubated with rabbit affinity purified anti-MICAL-L1 antibody followed by staining with Alexa Fluor 547-conjugated goat anti-rabbit antibody. Scale bar: 10 µm.

**Fig. 2.**
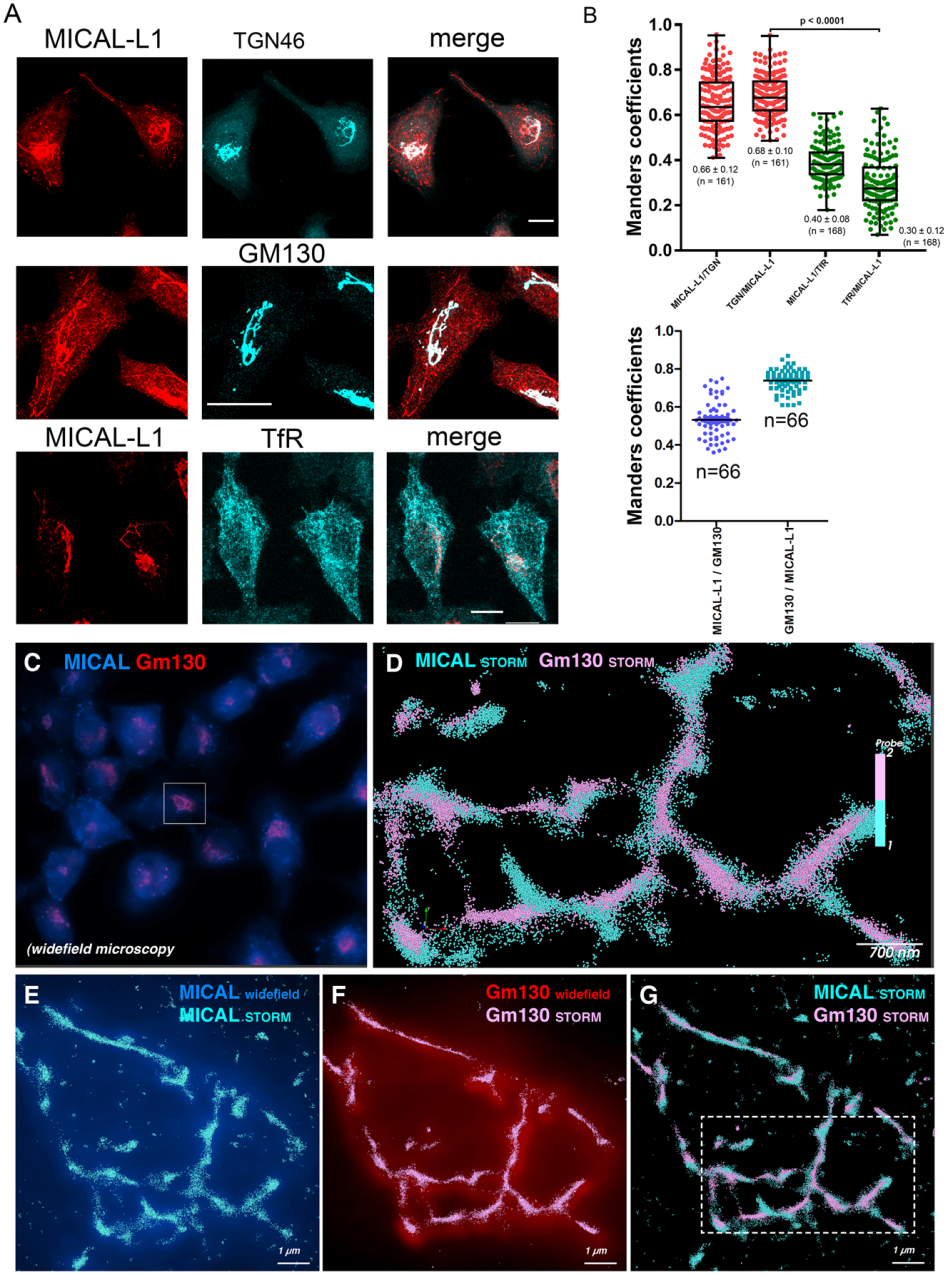
**Intracellular localization of MICAL-L1.** (A) Coverslips of HeLa cells were processed for immunofluorescence using rabbit affinity purified anti-MICAL-L1 and sheep monoclonal anti-TGN46, mouse monoclonal anti-GM130, and mouse anti-transferrin receptor (TfR) antibodies. 3D-projections of confocal images were collected. The projections were combined into a single color image in the third column (merge). Scale bars: 5 µm. (B) Quantification of MICAL-L1/TGN46, MICAL-L1/ GM130 and MICAL-L1/TfR colocalization was evaluated using coloc2 ImageJ plugin, *n*=numbers of cells analyzed are indicated on the figure. STORM Microscopy (C–G). HeLa cells labelled with anti-MICAL-L1-AlexaFluor647 and anti-GM130-AlexaFluor568. GA can be identified in wide-field microscopy (C) and then super-resolved using STORM (D–G). 3D STORM microscopy through 1 micron depth thanks to biplane module allowed visualization of single molecules MICAL-L1 (cyan sphere) and Gm130 (pink sphere). After several thousand pictures, 3D Golgi stacks can be reconstructed (G; magnified in D).

We used an affinity-purified polyclonal antibodies raised against the C-terminal domain (amino acid 520 to 863) to probe an immunoblot of cell extracts prepared from untransfected, Scramble (scr) and shRNAs targeting MICAL-L1 transfected HeLa cells. Anti-MICAL-L1 antibodies recognizes specifically a band of 130 kDa corresponding to the endogenous MICAL-L1 protein. The intensity of this band significantly decreased in MICAL-L1 silenced cells (∼70% depletion, Fig. 1A). In addition, the endogenous MICAL-L1 staining was significantly reduced in MICAL-L1-shRNA-treated cells as compared with mock-treated cells (Fig. 1B), thus further validating our antibody. We then determined MICAL-L1 intracellular localization in HeLa cells. Double labeling experiments with MICAL-L1 along with GM130 and TGN46, two markers of the GA, antibodies showed a significant colocalization of MICAL-L1 and Golgi markers (66±12%)**.** Although MICAL-L1 did not colocalize entirely with TGN46 and GM130, almost all TGN46 and GM130 staining colocalized with MICAL-L1 (72%). In addition, MICAL-L1 was also found in tubulo-vesicular structures in the cytoplasm. We then investigated MICAL-L1 distribution versus that of the transferrin receptor (TfR) which is mainly detected at steady state in early and recycling endosomes (RE)*.* Again MICAL-L1 partially colocalized with the TfR (40±8%; Fig. 2B,C).

The localization of endogenous MICAL-L1 by the Stochastic Optical Reconstruction Microscopy (STORM) was investigated by determining the position of each individual molecule independently as previously described (Lagache et al., 2018). The STORM achieves a spatial resolution at approximately 30 nm, and thus, enables the imaging of cellular structures with a much greater spatial resolution than the conventional wide-field microscope (Schermelleh et al., 2010; Sillibourne et al., 2011). Fixed HeLa cells were double stained with anti-MICAL-L1 and anti-GM130 antibodies. STORM images indicate that MICAL-L1 and GM130 exhibited a very close association in the perinuclear region of cells, suggesting that both proteins are associated with Golgi stacks (Fig. 2C,D,E–G for high magnification insets). However, the presence of GM130 in MICAL-L1 tubules was only found in a subset of cells (data not shown). We then performed double labeling with anti-MICAL-L1 and anti-TGN46 antibodies in 3D STORM microscopy and showed that, at the nanoscale level, both proteins are rather close but do not belong to the same cloud of molecule suggesting that they are close but in different Golgi cisternae (see Fig. S1,B,C for highest magnification).

### MICAL-L1 regulates the secretion pathway

The RUSH assay allows to synchronize the intracellular trafficking of cargoes fused to the streptavidin-binding peptide (SBP) upon addition of biotin in the cell culture medium. It monitors cargo secretion and their trafficking from Golgi to the cell surface. (Boncompain et al., 2012). We investigated the synchronous secretion of three membrane proteins, TNFα−SBP−mCherry (TNFα), E-cadherin-SBP-mCherry (cad), and GPI anchored-mCherry (GPI). These three proteins are transported through the biosynthetic pathway, from the ER to the Golgi and they are then delivered to the PM in HeLa cells (Boncompain et al., 2012). TNFα secretion in the culture medium is dependent on its cleavage by the TACE protease which is not expressed in HeLa cells. Thus, TNFα is not secreted and accumulates at the plasma membrane in HeLa cells.

**Fig. 3.**
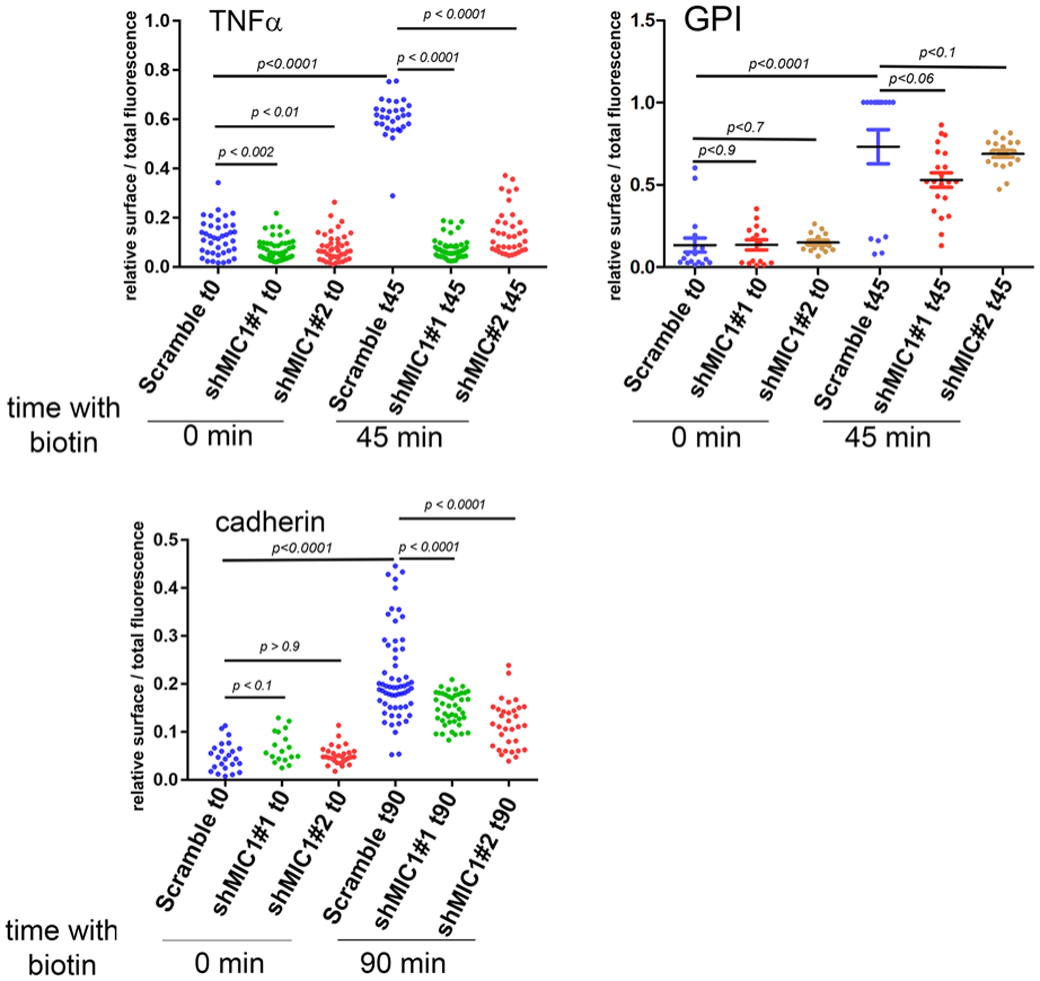
**MICAL-L1 inhibits cell surface delivery of TNFα and E-cadherin but not that of GPI.** HeLa cells expressing Scr and two different shRNAs targeting MICAL-L1 were transfected with TNFα-SBP-mCherry (TNFα), cadherin-mCherry (cadherin)t and GPI-mCherry (GPI) using the RUSH system. Cells were incubated with biotin at 37°C for 45 min for TNFα and GPI and 90 min for cadherin. Cells were washed with cold PBS at 0°C, Incubated with anti-mCherry at 0°C for 30 min, followed by another incubation with AlexaFluor647 donkey anti-rabbit for 30 min at 0°C to detect cargoes at the cell surface. Cells were then fixed and analyzed by confocal microscopy. The ratio of cell surface/total fluorescence was quantified, at least 40 cells for each condition were analyzed.

We quantified the release of TNFα, cad, and GPI transported to the cell surface in control and MICAL-L1 knockdown cells. TNFα, cad, and GPI localized at the plasma membrane were detected using the mCherry tags located at the extracellular domains with anti-mCherry antibodies without permeabilization and before PFA fixation. Although TNFα, and cad proteins reached the cell surface in control cells, shRNA depletion of MICAL-L1 impaired surface delivery of TNFα and to a lesser extent that of cad. The signal intensity of TNFα and cad at the cell surface deceased by ∼70% for TNFα and ∼40% for cadherin. In contrast, MICAL-L1 depletion did not affect the GPI cell surface delivery, suggesting that the role of MICAL-L1 in membrane trafficking is specific and restricted to a set of cargoes (Fig. 3).

**Fig. 4.**
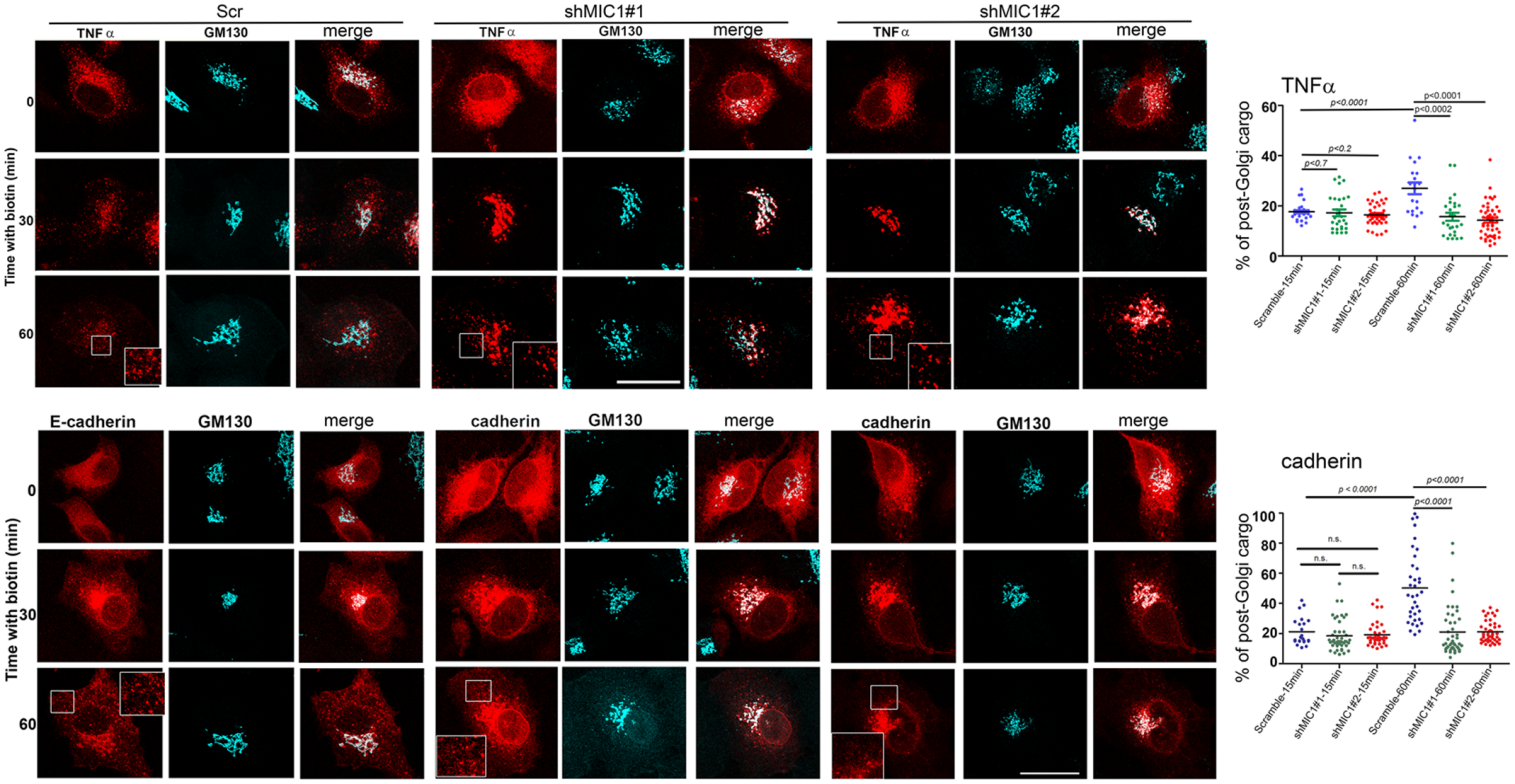
**MICAL-L1 controls membrane trafficking.** HeLa cells expressing Scr and two different shRNAs targeting MICAL-L1 were transfected with TNFα-SBP-mCherry, and cadherin-mCherry, using the RUSH system. After addition of 40 µM Biotin, cells were stained with anti-GM130 and analyzed by immunofluorescence at 0, 30 and 60 min. 3D-projections of images are shown. Note that depletion of MICAL-L1 delayed cargo transport to the PM. Scale bars: 10 µm. Quantification of post-Golgi vesicles was performed as indicated in the Materials and Methods section.

To confirm the inhibitory effect of MICAL-L1 on the delivery of membrane proteins, control and knock down cells expressing TNFα, and cad were analyzed by immunofluorescence at different time points after biotin addition. To quantify post-Golgi vesicles, we stained HeLa cells with the anti-GM130. As expected, before biotin addition, TNFα and cad were retained in the ER (Fig. S2). In control cells, both cargoes left the ER, reached a perinuclear region, and were then released in post-Golgi vesicles within 45–90 min after biotin addition. In contrast, both cargoes accumulated at the perinuclear region, and their PM delivery was inhibited in MICAL-L1 knockdown cells. We observed that TNFα and cad signals persist in the vicinity of the GA even after 30 min post-release in MICAL-L1 depleted HeLa cells. Quantification showed that the percentage of post-Golgi intensity of TNFα and cad spots was decreased (Fig. 4). The release of cargoes from the perinuclear zone was delayed in cells depleted of MICAL-L1.

HeLa cells depleted of MICAL-L1 and transfected with a siRNA-resistant GFP-MICAL-L1 construct were used to perform transport of TNFα in control and MICAL-L1 knockdown cells as indicated above. Interestingly, shRNA-resistant GFP-MICAL-L1 restored the transport of the TNFα to the PM when compared to the control thus ruling out off-target effects (Fig. S3, arrows). Our data suggests that MICAL-L1 plays an important role in the trafficking of TNFα and cadherin from the perinuclear zone to the PM.

We then examined the transport of TNFα-SBP−mCherry in live cells by confocal spinning-disk microscopy. In control cells, the synchronized trafficking of TNFα revealed a fast ER export; we detected scattered dots corresponding to ER exit sites. After biotin addition, TNFα accumulated in the perinuclear region within 10 min. After 15 min, post-Golgi TNFα cargoes appeared and PM staining became visible, whereas signal at the perinuclear region decreased to and finally almost completely disappeared after 40 min. In MICAL-L1 knockdown HeLa cells, TNFα carriers remained for long in the perinuclear region and their exit from this compartment was delayed as compared to control cells, further supporting that MICAL-L1 plays a key role in the transport to the cell surface (Movies 1–3).

### MICAL-L1-RBD may contribute to promote PACSINs mediated membrane deformation

**Fig. 5.**
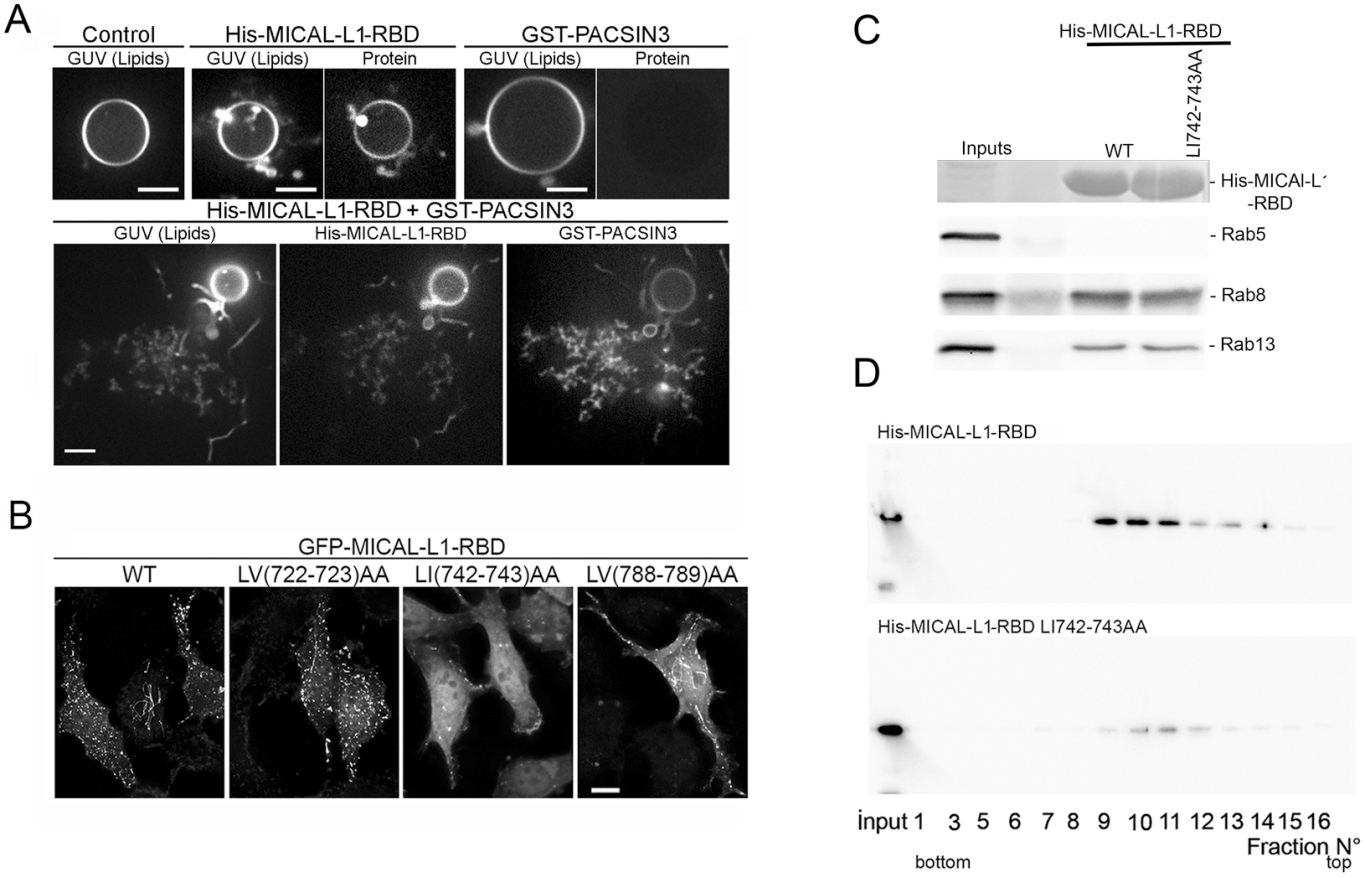
**Two amino acids in MICAL-L1-RBD domain are important for PA binding and association with membrane tubules.** (A) *In vitro* tubulation assays were performed with fluorescent PA-containing GUVs and His-MICAL-L1-RBD coupled to AlexaFluor 488 or GST-PACSIN3-AlexaFluor 647. After incubation, samples were imaged by video spinning confocal microscopy. Images were collected at 100-ms intervals (50-ms exposure per channel) using the same illumination and gain conditions. Images for GUV alone (control), GUV incubated with His-MICAL-L1-RBD, GST-PACSIN3, and His-MICAL-L1-RBD plus GST-PACSIN3. Scale bars: 5 µm. (B) Coverslips of HeLa cells expressing GFP-MICAL-L1-RBDWT, GFP-MICAL-L1-RBD LV (722-723) AA, GFP-MICAL-L1-RBD LI (742-743) AA and GFP-MICAL-L1-RBD LV (788-789) AA mutants were analyzed by immunofluorescence. 3D projections of images are shown. Scale bar: 10 µm. Note that mutations of LI742-743AA impairs MICAL-L1 tubulo-vesicular distribution. (C) Purified His-MICAL-L1 RBDWT and LI742-743AA bound to Nickel beads (coomassie staining) were incubated with HeLa cell extract to pulldown endogenous Rab5 (negative control), Rab8 and Rab13. Bound proteins were analyzed by immunoblotting using anti- Rab5, Rab8 and Rab13 antibodies. (D) Equal amounts of purified His-MICAL-L1-RBD WT and LI742-743 mutant proteins were incubated with liposomes enriched in PA, and then subjected to a flotation assay. Different fractions were collected and analyzed by blotting using anti-His for MICAL-L1 RBD detection. Note that Leu-Ileu742-743AA mutant reduces PA binding.

It was previously shown that MICAL-L1-RBD binds PA, and is required for its association with membrane tubules (Sharma et al., 2009). Although MICAL-L1-RBD domain does not display structural features known to deform membranes, we tested its ability to tubulate individual giant unilamellar vesicles containing PA (PA-GUV). No tubulation was observed in PA-GUVs in the absence of the RBD domain. However, we did detect few morphological changes (bubbles) on the surface of PA-GUV upon incubation of MICAL-L1-RBD (Fig. 5A). Given that MICAL-L1 interacts with PACSINs, proteins with BAR domains (Giridharan et al., 2013), we investigated the capacity of both proteins to deform PA-GUVs. Upon addition of fluorescently labeled GST-PACSIN3 (1 µM) to GUVs, no protein was observed to bind to GUVs and no tubules were apparent. However, when His-MICAL-L1-RBD and GST-PACSIN3 were co-incubated with GUVs, localized clusters of tubules formed and elongated on the surface of GUVs, forming a membrane network (Fig. 5A). GST-PACSIN3 was observed to be associated mostly with tubules and weakly with GUVs, suggesting greater affinity for tubules with a high curvature. A similar pattern of tubulation was observed by Takiguchi et al. for PACSIN2 on GUVs (Tanaka-Takiguchi et al., 2013). These data suggest that MICAL-L1 promotes PACSINs dependent membrane tubulation.

### Two amino acids at the MICAL-L1 C-terminus are important for PA binding and association with membrane tubules

The RBD sequence analysis exhibits hydrophobic amino acids clusters (Fig. S4A). Substitutions of Leu-Val at positions 722-723 and 788-789 by Ala-Ala in MICAL-L1RBD did not alter MICAL-L1-RBD tubulo-vesicular distribution. However, mutations of Leu-Ileu at positions 742-743 to Ala-Ala significantly reduced MICAL-L1 association with tubulo-vesicular structures (Fig. 5B). As MICAL-L1-RBD contains structural elements required for the interaction with PA and with GTP-bound Rab8 and Rab13 (Abou-Zeid et al., 2011; Sharma et al., 2010; Zahraoui, 2014), experiments were performed to determine whether or not Leu-Ileu 742-743 mutations interfered with PA or Rab binding. MICAL-L1-RBD WT and LI742-743AA-RBD mutant were expressed as His-fusion proteins, purified, and used to pull down endogenous Rab8 and Rab13 from HeLa cell lysates. Rab5 was used as a negative control since it does not interact with MICAL-L1. Fig. 5C shows that His- MICAL-L1-RBDWT and mutant bound Rab8 and Rab13, but not Rab5, indicating that LI742-743 mutations did not abolish the binding of MICAL-L1-RBD to Rab8 and Rab13. To verify whether or not these mutations affected the binding of MICAL-L1-RBD to PA, liposome flotation assays were performed. Equal quantities of purified His-MICAL-L1-RBDWT and LL742-743AA mutant proteins were incubated with rhodamine-labeled liposomes under physiological conditions. Compared to the wild-type RBD, mutations of Leu-Ileu742-743 significantly reduced MICAL-L1-RBD binding to PA (Fig. 5D; Fig. S5). Our results suggest that Leu-Ileu742-743 were critical for both MICAL-L1 association with PA and with membrane tubules. Interestingly, expression of MICAL-L1 mutant LI742-743AA, which is unable to bind PA, did not restore the transport of TNFα. Compared to Scr cells, we observed that TNFα staining was accumulated in perinuclear regions and was not detected in scattered cytoplasmic vesicles in MICAL-L1 knockdown cells expressing MICAL-L1LI744AA mutant, suggesting that PA binding is required for membrane trafficking towards the cell surface (Fig. S4B, arrows). These results are in agreement with previous studies highlighting a critical role for PA in membrane trafficking (Giridharan et al., 2013).

## DISCUSSION

Using our affinity purified antibody, we showed that MICAL-L1partially colocalized with GA as well as with RE markers. The localization of MICAL-L1 in Golgi structures has not been reported in previous studies (Etoh and Fukuda, 2019; Sharma et al., 2009). Our affinity purified antibodies were raised against the C-terminal 343 amino acids containing part of the PRD and RBD domain of MICAL-L1. The affinity purified antibody used here was specific because it stained a single band of the expected MW. Furthermore, the intensity of the band in the blot and immunofluorescence staining of the endogenous protein decreased significantly in shRNA depleted MICAL-L1 HeLa cells. Our antibody thus appeared to specifically recognize MICAL-L1. STORM imaging suggests that MICAL-L1 pattern appeared intermingled to GM130 structures and close to TGN46 labelled structures. Further investigations would be needed to check whether MICAL-L1 labeled structures could reside within the trans-Golgi compartment.

We then analyzed the first wave of secretion of transport carriers at physiological temperature using the RUSH assay in HeLa cells. We found that MICAL-L1 is required for cargo delivery to the plasma membrane. Recent findings revealed that the GA and RE are functionally overlapping. In HeLa cells, REs are interconvertible between a Golgi-associated REs and free REs. Detachment/attachment of REs and Golgi stacks are continuously observed, suggesting the existence of structural and functional relationships between REs and GA (Fujii et al., 2020). Therefore, Golgi/REs may constitute an overlapping hub connecting endocytosis and exocytosis. Our data suggest that in addition to its role in the recycling of internalized cargoes (Sharma et al., 2009), MICAL-L1 may also regulate the exocytosis/recycling of biosynthetic cargoes. We showed that MICAL-L1 is required for cell surface transport of both TNFα and E-cadherin, two membrane proteins transported from the GA to the PM via REs (Lock and Stow, 2005; Srivastava and Lacy, 2014). Interestingly, the interaction of MICAL-L1 with Rab8 and 13 involved in post-Golgi trafficking (Abou-Zeid et al., 2011; Huber et al., 1993; Nokes et al., 2008; Sharma et al., 2009) strengthens the notion that MICAL-L1 may also be involved in post-Golgi trafficking. Several lines of evidence suggest that multiple classes of cargos are sorted before being delivered to the plasma membrane (De Matteis and Luini, 2008). We observed that the time of residence in the Golgi and exit from the GA of cargoes was different. A cargo such as TNFα exits very quickly, while E-cadherin stays for a longer time in the GA, suggesting intra-Golgi segregation as previously reported (Boncompain et al., 2012). Importantly, MICAL-L1 was not required for trafficking of GPI-anchored protein (GPI-APs). In epithelial cells, GPI-APs are delivered in specific secretory vesicles from the TGN to the apical, but not to the basolateral membrane. The formation of GPI-AP oligomers takes place at the GA, and it has been suggested that oligomerization facilitates GPI-AP segregation from other secretory proteins at the TGN (Paladino et al., 2004). Therefore, TNFα/E-cadherin and GPI-AP carriers are sorted and routed along two different post-Golgi pathways. We propose that MICAL-L1 is involved in the sorting of membrane proteins that are routed to the PM via the GA/RE overlapping compartment. It should be interesting to investigate where the TNFα/Cadherin and GPI start to segregate and the potential role of MICAL-L1 in this sorting. It has been reported that TfR and Lamp1 segregate during the early stage of Golgi transport (Chen et al., 2017), and similar segregation might occur between TNFα/Cadherin and GPI. Studies, using RUSH for TNFα and GPI might, therefore, reveal their sorting mechanisms.

The C-terminal coiled-coil domain of MICAL-L1 is required for PA binding. The MICAL-L1 C-terminal directly binds to PA. Substitution of two residues, Leu-Ileu 742-743 by Ala-Ala, impairs both the interaction of MICAL-L1-Cter with PA and the targeting to tubulo-vesicular structures. Compared to the wild-type RBD, mutations of Leu-Ileu742-743 significantly reduced MICAL-L1-RBD binding to PA, suggesting that additional residues were also implicated. Indeed, two KR residues at positions 851-852 are required for optimal membrane association (Sharma et al., 2009)**.** It has been previously shown that a stretch of hydrophobic residues at positions 721-726 were critical for MICAL-L1 tubule association. It is possible that LV722-723 mutations alone are not sufficient, but larger region (721-726) is necessary for MICAL-L1 association with tubules (Sharma et al., 2009).

Although MICAL-L1-Cter domain does not encompass any obvious amphipathic helical sequence or displays homology to BAR domains, we show that it is able to promote PACSIN mediated deformation of GUVs *in vitro*. Surprisingly, PACSIN3 alone (at least under our experimental conditions) is not able to induce tubulation of GUVs, suggesting a high energy barrier for its oligomerization and induction of membrane tubulation, also reported for PACSIN2 (Tanaka-Takiguchi et al., 2013).

Our data and others (Sharma et al., 2009; Giridharan et al., 2013) suggest that PA is a key component for the recruitment of MICAL-L1 to membrane tubules. PA has been implicated in membrane fission, suggesting that it may promote the release of tubulo-vesicular carriers (Jovanovic et al., 2006). We speculate that MICAL-L1 scaffold couples membrane shaping components such as PACSINs, Rabs and cargo sorting to PA microdomains, which may facilitate membrane sorting/targeting during cargo delivery to the plasma membrane. Interestingly, Rab10, a partner of MICAL-L1, has been involved in the regulation of tubular endosome formation through KIF13A and KIF13B motors (Etoh and Fukuda, 2019). Thus, MICAL-L1 in a complex with its interacting proteins might facilitate membrane deformation and contribute to the formation of post-GA/RE carriers in the secretory pathway.

## MATERIALS AND METHODS

### Constructs

Cloning of MICAL-L1 into pEGFP-C1 and mCherry vectors were previously described (Abou-Zeid et al., 2011). All the RUSH plasmids used in this study, use streptavidin-KDEL as a hook. Briefly, the hook (streptavidin-tagged protein) allows retention of the SBP-tagged cargo in the ER in the absence of biotin thanks to streptavidin–SBP interaction (Boncompain et al., 2012). The release of the RUSH cargoes was induced by addition of 40 µM of D-biotin (Sigma-Aldrich).

### Lipids reagents

All reagents and *β*-casein from bovine milk (>99%) were purchased from Sigma-Aldrich (Sigma-Aldrich, France). DOPC (1,2-dioleoyl-*sn*-glycero-3-phosphatidylcholine), DOPS (1,2-dioleoyl-*sn*-glycero-3-phospho-L-serine), DOPE (1,2-dioleoyl-*sn*-glycero-3-phosphatidylethanol- amine), Egg PA (L-α-phosphatidic acid) and Egg Rhod PE (L-α-phosphatidylethanolamine-N-(lissamine rhodamine B sulfonyl) were purchased from Avanti Polar Lipids, Inc. (Avanti Polar Lipids, USA). Stock solutions of lipids in chloroform (10 mg/ml) were stored at −20°C in amber vials (Sigma-Aldrich, France). Lipid stock solutions were mixed to achieve the desired molar ratio of DOPC/DOPE/DOPS/Egg PA/Egg Rhod PE (33/33/13/20/0.8) at a total concentration of 1 mg/ml in chloroform. After use, argon was added to vials before sealing with paraffin film (Parafilm, USA) to prevent lipid oxidation.

### Mutagenesis

For mutagenesis of hydrophobic amino acids at the Cter of MICAL-L1, QuickChange site-directed mutagenesis kit (Agilent Technologies Inc., Santa Clara, CA, USA) was used. Substitutions of amino acids were performed according to the manufacturer's instructions. Oligonucleotides for mutagenesis of LV722-723 to AA, oligo sens, 5′-GAGCTTGAACCAGTCCGCCGCCATGTCATCCTCACGG-3′; oligo anti-sens, 5′-CCGTGAGGATGACATGGCGGCGGCTGGTTCAAGCTC-3′. For mutagenesis of LI722-723 to AA, oligo sens, 5′-TGCTGCTTGAAGACATAGGCGGCCTCGGACTCTCGCCGCAC-3′, oligo anti-sens, 5′-GTGCGGCGAGAGTCCGAGGCCGCCTATGTCTTCAAGCAGCA-3′. For mutagenesis of LV788-789 to AA, oligo sens, 5′-CTGCTCAATGAGGGTCGCAGCCTCCTGCATCAGCACC-3′, oligo anti-sens, 5′-GGTGCTGATGCAGGAGGCTGCGACCCTCATTGAGCAG-3′. All mutations were verified by sequencing.

### Antibodies

Purified His-MICAL-L1-RBD protein (amino acids 520-863 of MICAL-L1) was injected into rabbits to generate polyclonal antibodies (Covalab. Villeurbanne, France). The resulting antiserum was affinity purified against His-MICAL-L1RBD protein. anti-mCherry and anti-Histidine (His) rabbit polyclonal antibodies were from Roche (Basel, Switzerland), anti-GM130 monoclonal antibody (BD Transduction laboratory, CA, USA), anti-human TGN46 (Bio-Rad, CA, USA),anti-trasferrin receptor (Thermo Fisher Scientific, Rockford, IL, USA), anti Rab5, Rab8 from Transduction laboratories (USA) and Rab13 from Sigma-Aldrich (USA) and donkey-affinity purified secondary antibodies conjugated to AlexaFluor 488, 568, and 647 were from Jackson ImmunoResearch Laboratories (West Grove, PA, USA). The protein disulphide-isomerase (PDI) mouse monoclonal antibody from Enzo Life Sciences (France).

### Cell culture and transfection

HeLa cells (ATCC CCL-2) were grown in DMEM containing 10% fetal calf serum (FCS) (Gibco, Watham, MA, USA) supplemented with 10% fetal calf serum, 2 mM glutamine, 100 U/ml penicillin, and 10 mg/ml streptomycin. The cells were incubated at 37°C under a 5% CO_2_ atmosphere. Stable HeLa cells expressing shRNA of MICAL-L1 were generated. Positive clones were selected in the same medium supplemented with 0.3 µg/ml zeocin (Gibco, Waltham, MA, USA). Stably transfected clones were maintained under selection in 0.1 µg/ml of zeocin. The shRNA sequences that efficiently inhibited proteins expression were as follow: MICAL-L1 shRNA oligo-sens, 5′-ACCTCGTGGAGCCTAGAGTGGAACAATC AAGAGTTGTTCCACTCTAGGCTCCACTT-3′; MICAL-L1 shRNA oligo-antisens, 5′-CAAAAAGTGGAGCCTAGAGTGGAACAACTCTTGATTGTTCCACTCTAGGCTCCACG-3′; PACSIN3 shRNA oligo-sens, 5′-ACCTCGGCTTGTTCTAGCGTGTATTATCA AGAGTAATACACGCTAGAACAAGCCTT-3′; PACSIN3 shRNA oligo-antisens, 5′-CAAAAAGGCTTGTTCTAGCGTGTATTACTCTTGATAATACACGCTAGAACAAGCCG-3′. HeLa cells were transfected with Lipofectamine 3000 according to the manufacturer's protocol (Invitrogen, Grand Island, NY, USA).

### Immunoblot

HeLa cells were lysed in buffer, 20 mM Tris HCl pH 7.5, 150 mM NaCl, 0.5% NP-40 with a protease cocktail inhibitor (Sigma-Aldrich). Solubilized material was recovered by centrifugation at 18.000 ***g*** for 15 min at 4°C and supernatants were collected. Protein amounts were determined using the Pierce BCA assay (Life Technologies, PA, USA) and equal quantities of proteins were separated by SDS-PAGE and transferred electrophoretically to nitrocellulose filters. Immunoblots were performed using anti-MICAL-L1 antibodies and enhanced chemiluminescence according to the manufacturer's protocol (Thermo Fisher Scientific, Rockford, IL, USA).

### GST pull-down assay

The cDNA encoding His-MICAL-L1-RBD (amino acids 520-863) WT or LI742-743AA was inserted in a pET15b expression vector using NdeI-XhoI restriction sites. The His-MICAL-L1-RBD fusion proteins were produced in *E. coli* and purified on Ni2-beads It were then incubated with HeLa cell extract for 4 h at 4°C, washed and bound material was analyzed by SDS-PAGE and immunoblotting using anti-Rab5, Rab83 and Rab13 antibodies.

### Liposome flotation assay

Liposomes were prepared with a mass ratio composition of 87% POPC, 3% Lissamine rhodamine phosphatidyl ethanolamine and 10% of POPC, POPA or POPS in Hepes/NaCl buffer (25 mM Hepes pH 7.3, 150 mM NaCl). 800 nM of GST-PACSIN3 or His-MICAL-L1-Cter purified proteins were incubated with 500 µM of each liposome preparation for 30 min at room temperature. Samples were adjusted to 55% sucrose and loaded at the bottom of a Beckman SW55 Ti centrifugation tube. Samples were then overlaid by a discontinuous sucrose gradient (50%, 40%, 30%, 20%) and Hepes/NaCl buffer was added on the top of the tube. Liposomes were centrifuged at 150,000× ***g*** for 4 h at 4°C. Fractions were collected from the top and separated by SDS-PAGE and analyzed by immunoblot using rabbit polyclonal anti-His antibodies to detect MICAL-L1 RBD.

### Preparation of giant unilamellar vesicles (GUVs)

GUVs were prepared by the eletroformation method using conducting Indium Tin Oxide coated glass slides (ITO, Präzisions glas & optik GmBH, Germany) (Mathivet et al., 1996; Meleard et al., 2009; Morales-Penningston et al., 2010). A lipid solution of 10 µl was deposited on ITO slides by using a 5 µl Hamilton syringe to make a dry lipid film as thin as possible. The lipid coated ITO slides, assembled with sigillum wax (Vitrex, Denmark), were dried under vacuum for 45 min at room temperature. The lipid films were then hydrated with a sucrose/Tris growth buffer (100 mM of sucrose and 10 mM of Tris, at pH 7.4) and sinusoidale AC current at 1V (peak to peak) with a frequency of 10 Hz was applied for 45–90 min at room temperature. GUVs were extracted by pipetting directly from GUV-rich regions from the formation chamber. Collected GUVs were then transferred in observation buffer (70 mM of NaCl and 10 mM of Tris, at pH 7.4) with an osmolarity of 10–20 mOsm higher than that of the growth buffer.

### Protein-membrane binding assay

Observation chambers were prepared using 60×24 mm coverslips (Menzel-Gläser, Germany) and 40×22 mm coverslips (VWR International, France). Before use, the chambers were passivated with a 5 mg/ml *β*-casein solution (100 mM NaCl and 10 mM Tris, at pH7.4) for 15–30 min to prevent GUVs from adhering to the glass surface. Chambers were then rinsed several times and filled with observation buffer. GUVs were incubated with either MICAL-L1-RBD at 1 µM, or GST-PACSIN3 at 1 µM in the observation buffer, or with both of them simultaneously. The protein was allowed to bind to GUVs for 20–40 min on ice before observation. GUVs were observed with a spinning disk confocal microscope inverted Nikon Eclipse T*i*-E microscope with 100x oil objective. Images were recorded with an EM-CCD Evolve camera. The exposure time for all images was 50 ms

### Immunofluorescence staining

HeLa Cells on coverslips were fixed with 4% paraformaldehyde for 15 min at room temperature and permeabilized using 2% BSA and 0.1% Triton X100 for 15 min at room temperature in PBS. Cells were then incubated with primary antibody in 2% BSA, 0.1% triton X100 in PBS for 1 h at room temperature and then incubated with secondary conjugated antibody for 30 min at room temperature in the same buffer. After washing, samples were mounted in prolong (eBioscience). Alexa Fluor 488-transferrin pulse-chase assays were done as described previously (Jović et al., 2009).

### Fixed cell confocal imaging

Image acquisition was performed on an inverted confocal microscope (Leica DMI6000) with a 63x (1.4 NA) objective and a MicroMAX camera (Princeton Instruments) or ORCA Flash4.0 (Hamamatsu). Z stack of 7-10 plans (0.4 microns step) were acquired using Metamorph software (Molecular Devices, Sunnyvale, CA, USA). Images were then generated by compiling three-dimensional maximum intensity projections of plans using ImageJ software.

To evaluate protein colocalization we used Coloc 2, a pre-installed plugin on FIJI. We employed a bisection threshold regression on region of interests corresponding to single cell masks. Numerical correlation parameters such as Mander's coefficients are recorded as well as the 2D intensity histogram.

For transport of TNFα-mCherry, cadherin-mCherry, and GPI-mCherry to the cell surface, cells were washed at 0°C with PBS. Proteins at the cell surface were stained with anti-Cherry antibodies at 0°C for 60 min followed by incubation at 0°C with Alexa 746 donkey anti-rabbit secondary antibodies. Cells were then fixed with 4% PFA and analyzed. Cherry fluorescence gave the total amount of proteins expressed in the cell. The ratio of cell surface over total fluorescence was calculated. Experiment were performed in triplicate and more than 50 cells per experiment were quantified.

### Image quantification

To quantify the ratio of cargo transported to the surface, we first segmented cell morphology using the GFP channel. Background removal was performed by globally subtracting the mean fluorescent intensity of a region outside cells. The generated cell mask was then used to measure the mean fluorescence intensity of cargoes at the surface and within the cell. *n*=number of cells quantified is indicated in the figure.

To quantify the amount of cargoes exiting the Golgi (post-Golgi vesicles) we both segmented endogenously GFP-expressing cell and GM130-labelled Golgi morphology. We performed a rolling ball of 50 pixels to remove background signal. We then measured the cargo fluorescence intensity in the entire cell without taking into account the Golgi contribution

### Statistical significance test

Statistical tests used were unpaired nonparametric tests such as *t*-test and Kruskal–Wallis followed by a post hoc Dunn's test.

### Live cell imaging setup – Spinning disk

HeLa cells were seeded onto 18 mm-diameter glass coverslips, 1 day before trans­fection. Twenty hours after transfection with the TNFα-SBPEGFP RUSH plasmid (Boncompain et al., 2012), coverslips were transferred into a Chamlide chamber, filled with pre-warmed DMEM medium (Invitrogen). At time 2 min, medium was removed and D-biotin (Sigma-Aldrich) at 40 μM final was introduced in the chamber. Time-lapse acquisitions were done at 37°C in a thermostat-controlled chamber. Fluorescent images were sequentially acquired every 40 s for 60 min using a HCX APO 1.3 glycerol 63 X objective and MetaMorph software (Molecular Device). We used a LEICA DMI8 microscope (LEICA MICROSYSTEMS) equipped with a CSU-X1 spinning-disk confocal unit (Yokogawa Electric Corporation) and an ORCA -Flash4.0 V3 Digital sCMOS camera (Hamamatsu Photonics) in a controlled environment box (37°C and 5% CO2, PECON). The microscopy system was equipped with a laser combiner (Errol) comprises of a 488 nm (KVANT) and a 561 nm (Oxxius LBX) laser line. GFP (resp. mCherry/mRFP) emission light were collected with a stringent single bandpass filter 525/50-25 (resp. 620/60-25). The microscopy system was driven by Metamorph (Molecular Devices).

### 3D-STORM microscopy

HeLa cells were seeded on 18 mm #1.5 MENZEL glaser coverslips previously cleaned with plasma cleaner and coated with poly-Ornithine. Cells were fixed with 4% PFA during 10 min, permeabilized with 2% BSA-PBS-Triton X100 0.1% for 15 min at room temperature. Cells were then incubated with Rabbit anti-MICAL-L1 and mouse anti-GM130 antibody in 2% BSA, 0.1% triton X100 in PBS for 36 h at 4°C. After washing, cells were then incubated with donkey anti-rabbit-Alexfluor647 and donkey anti-mouse Alexafluor568 secondary conjugated antibodies for 2 h at room temperature in the same buffer. After washing with PBS, Hela cells were fixed in PBS- 4%PFA and 0.2% glutaraldehyde for 10 min. Cells were washed with PBS, mounted in an alveolar slide with dental silicon and analyzed with Bruker Optera/Vutara microscope as previously reported (Lagache et al., 2018). Samples were imaged in a photoswitching buffer containing 100 mM MEA and oxygen scavenging system (0.5 mg/ml glucose oxidase, 40 mg/ml catalase, 10% glucose) in PBS. They were excited with 640 and 561 laser lines and with 405 laser line. 3D-STORM images were reconstructed from a series of 10000 frames as previously reported (Collot et al., 2019) using Bruker Srx software.

## Supplementary Material

Supplementary information
