# Supplementary material for: MICAL-L1 is required for cargo protein delivery to the cell surface
Source: Biol Open. 2021 Jun 8;10(6):bio058008. doi: 10.1242/bio.058008 (PMC8214422; doi:10.1242/bio.058008)
Supplement: Supplementary information [file biolopen-10-058008-s1.pdf]

Fig S1

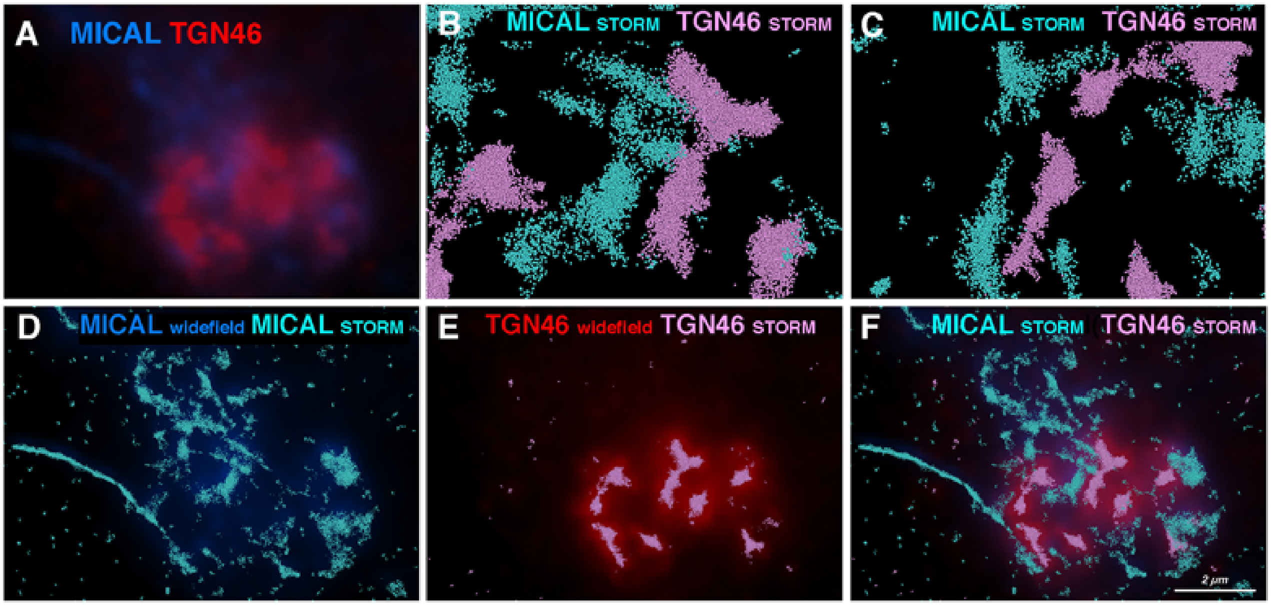

Figure S1, STORM Microscopy HeLa cells labelled with anti-MICAL-L1- and anti-TGN46 antibodies. In wide-field microscopy (A) MICAL (in dark blue) seems to be close to TGN46 positive structures (in red). However, when switching to super-resolved pictures using 3D STORM microscopy (B-F) to unravel individual molecules localization, we can see that TGN46 molecules (pink spheres) are distant in 3D from MICAL-L1 (Cyan spheres, in F) and are part of distinct clouds of proteins as can be seen at high magnification in B and C.

Biology Open • Supplementary information

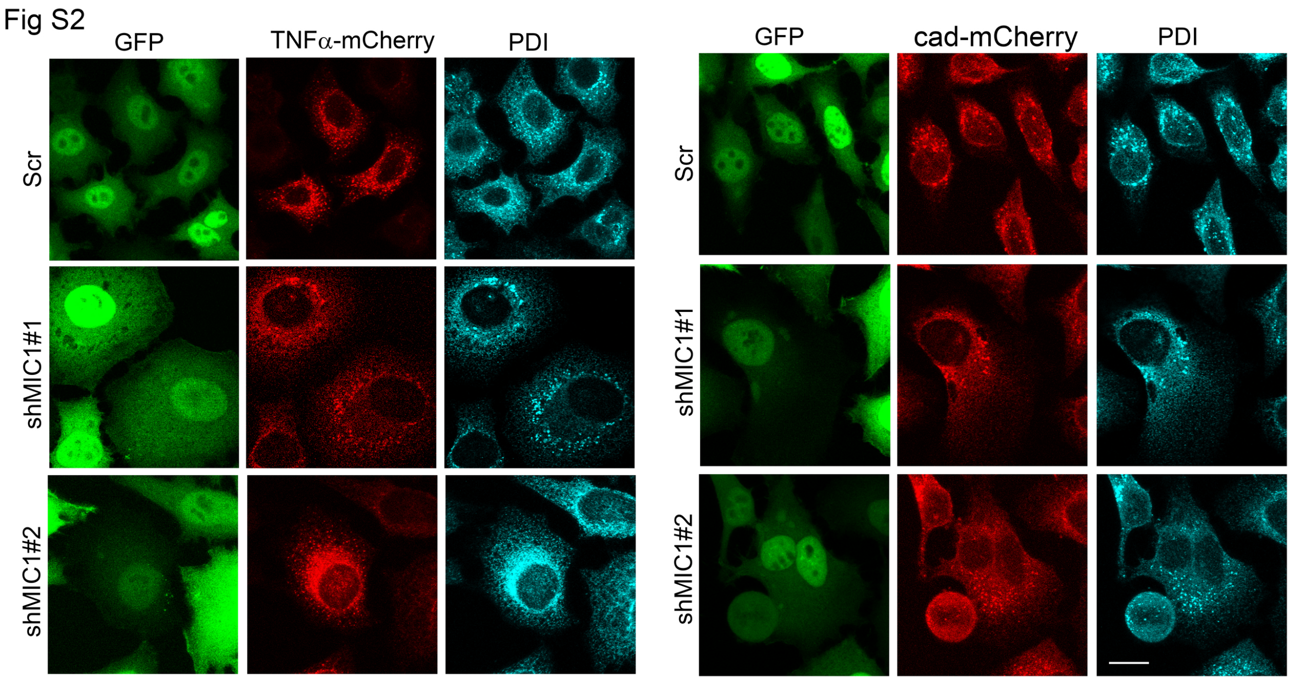

Figure S2, Coverslips of Scr and MICAL-L1 shRNA depleted cells transfected with TNFα-mCherry or Cadherin-mCherry were PFA fixed, stained with anti-PDI (protein disulphide-isomerase) and analyzed by immunofluorescence confocal microscope. Without addition of biotin, TNFalpha and Cadherin cargoes accumulate at the ER costaind with PDI.

Biology Open • Supplementary information

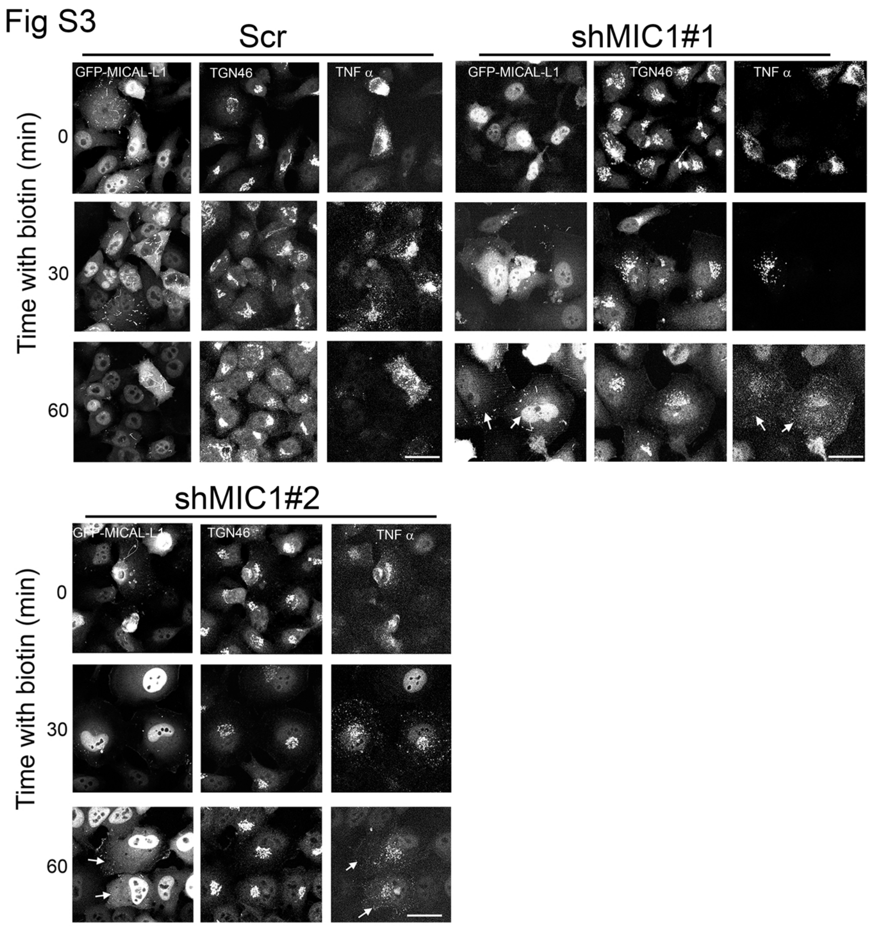

Figure S3, (A) Coverslips of control and MICAL-L1 depleted cells were cotransfected with TNFα-mCherry and shRNA-resistant GFP-MICAL-L1 using the RUSH system and analyzed as indicated in figure1. Cells were costained with anti-TGN46. Note that in cells expressing shRNA-resistant GFP-MICAL-L1 (arrows) detected as tubulo-vesicular structures, TNFα-mCherry cargoes were observed in post Golgi vesicles 30-60 min after addition of biotin, suggesting that re-expression of shRNA-resistant GFP-MICAL-L1 restored the transport of the TNFα to the PM.

Fig S4

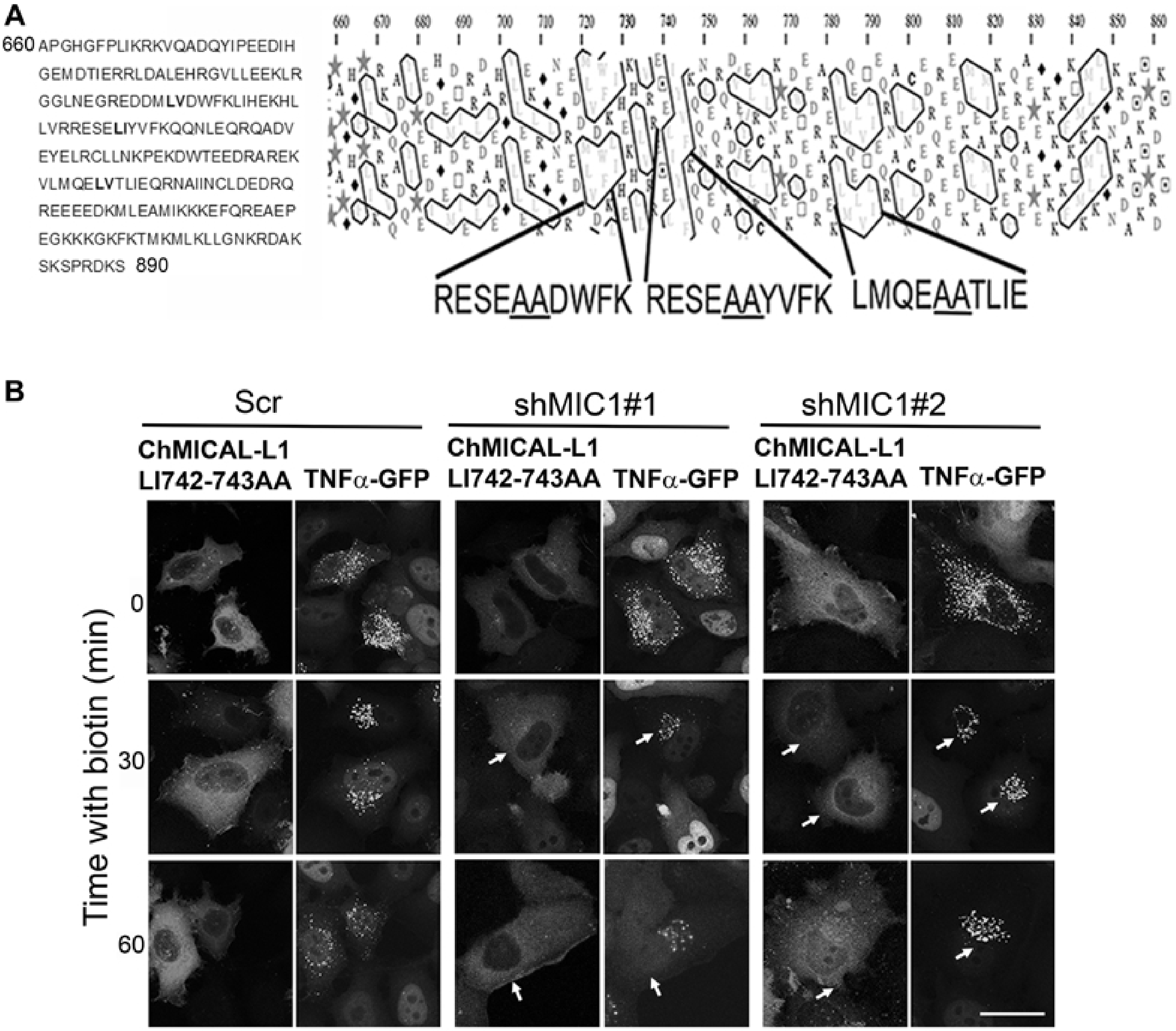

Figure S4, (A) amino acid sequence at positions 660-890 of MICAL-L1-RBD is presented and hydrophobic residues LV722-723, LI742-743 and LV788-789 are in bold. Hydrophobic Clusters Analysis (HCA) was performed using the web site, http://mobyle.rpbs.univ-paris-diderot.fr/cgi-bin/portal.py?form=HCA#forms::HCA. HCA of MICAL-L1 RBD revealed the presence of hydrophobic residue clusters. Mutations of LV at position 722-723 to AA, LI at position 742-743 to AA and LV at position 788- 789 to AA were underlined. (B), expression of mCherry-MICAL-L1LI742-743AA (PA mutant) did not restore the transport of the TNFα cargo to the plasma membrane. Coverslips of control and MICAL-L1 depleted HeLa cells were cotransfected with TNF α-EGFP and mCherry-MICAL-L1LI742-743AA mutant using the RUSH system and analyzed as indicated in figure1. Note that in cells expressing (PA mutant) detected as a diffuse staining (arrows), TNFα cargoes were accumulated in perinuclear regions within 30-60 minutes after addition of biotin and were not detected in scattered cytoplasmic vesicles. Bar 10 μm.

Biology Open • Supplementary information

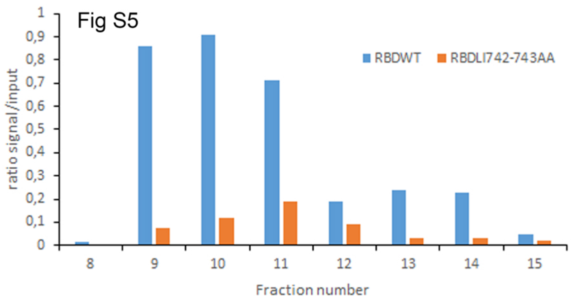

FigureS5. Quantification of the experiment performed in Figure 5D. After blotting, His-RBD bands were quantified using the ImageJ program and Microsoft Excel software. It did not seem to show a change in the flotation assay of the mutant compared to the wild type. Mutations of Leu-Ileu742-743 significantly reduced MICAL-L1-RBD binding to PA.

Biology Open • Supplementary information

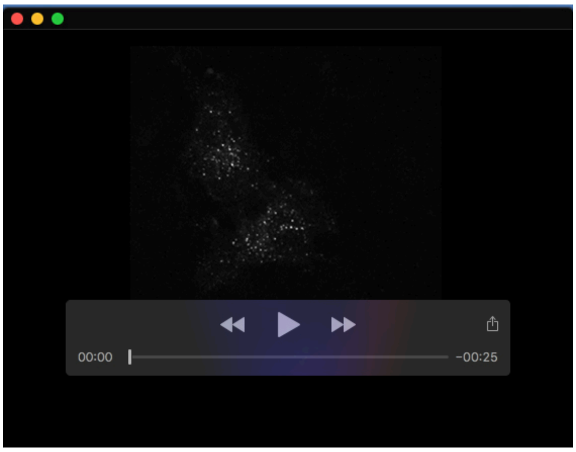

Movie 1

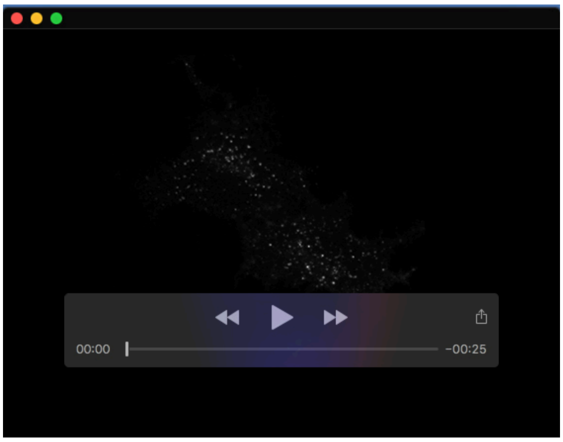

Movie 2

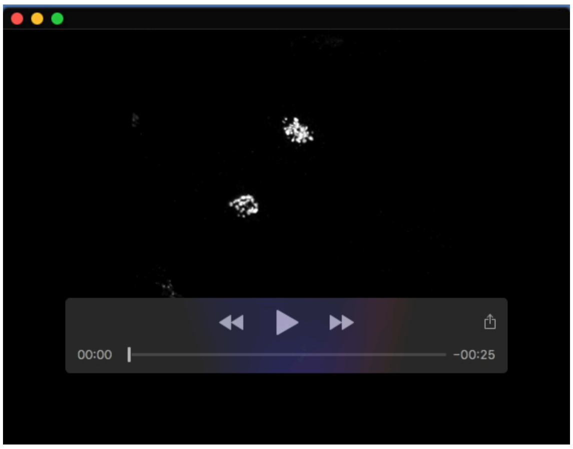

Movie 3

Biology Open • Supplementary information

Movie 1-3, MICAL-L1 controls TNF traffic between Golgi and plasma membrane. HeLa stable cell lines expressing Scramble and 2 different shRNA fused to GFP targeting human MICAL-L1 were transfected with TNFα-SBP-mCherry using the RUSH system. Cells were then imaged using a spinning disk confocal microscope at 37 °C. Before biotin treatment, we detected TNFα-SBP-mCherry as scattered dots corresponding to ER exit sites. After biotin addition, TNFα-SBP-mCherry was observed in the Golgi complex within 10 min. After 15 min, post-Golgi vesicles appeared and plasma membrane staining became visible whereas signal at the crossed the perinuclear region decreased disappearing almost completely after 40 min. TNFα proteins crossed the perinuclear region in 10–15 min on average in control cells whereas in MICAL-L1 depleted cells, TNFα cargoes were delayed and crossed the crossed the perinuclear region in 30 min on average. Movies are presented as single color movies of TNFα-SBP-mCherry.
